# Using several pair-wise informant sequences for *de novo *prediction of alternatively spliced transcripts

**DOI:** 10.1186/gb-2006-7-s1-s8

**Published:** 2006-08-07

**Authors:** Paul Flicek, Michael R Brent

**Affiliations:** 1 European Bioinformatics Institute, Wellcome Trust Genome Campus, Hinxton, Cambridge CB10 1SD, UK; 2 Laboratory for Computational Genomics, Washington University, Saint Louis, MO 63130, USA

## Abstract

**Background:**

As part of the ENCODE Genome Annotation Assessment Project (EGASP), we developed the MARS extension to the Twinscan algorithm. MARS is designed to find human alternatively spliced transcripts that are conserved in only one or a limited number of extant species. MARS is able to use an arbitrary number of informant sequences and predicts a number of alternative transcripts at each gene locus.

**Results:**

MARS uses the mouse, rat, dog, opossum, chicken, and frog genome sequences as pairwise informant sources for Twinscan and combines the resulting transcript predictions into genes based on coding (CDS) region overlap. Based on the EGASP assessment, MARS is one of the more accurate dual-genome prediction programs. Compared to the GENCODE annotation, we find that predictive sensitivity increases, while specificity decreases, as more informant species are used. MARS correctly predicts alternatively spliced transcripts for 11 of the 236 multi-exon GENCODE genes that are alternatively spliced in the coding region of their transcripts. For these genes a total of 24 correct transcripts are predicted.

**Conclusion:**

The MARS algorithm is able to predict alternatively spliced transcripts without the use of expressed sequence information, although the number of loci in which multiple predicted transcripts match multiple alternatively spliced transcripts in the GENCODE annotation is relatively small.

## Background

Accurate prediction of protein-coding genes in mammals remains a challenging and active area of research [1]. In the past decade the most important advance in *de novo *gene prediction came with the initial availability of extensive human and mouse genomic sequences. Several gene prediction algorithms were introduced at that time that improved gene prediction by using the specific patterns of evolutionary conservation that are indicative of protein coding genes [2-4].

### Dual-genome gene finding algorithms

All of the dual-genome (category 4) gene finders participating in EGASP rely on alignments to one or more informant genome sequences. For predicting human genes, dual-genome gene prediction algorithms most often use the mouse genome sequence as a source of evolutionary conservation information. This was originally a consequence of the early availability, with respect to other mammals, of the mouse genome sequence [5-8]. However, as additional genomes were sequenced, it became apparent that the evolutionarily divergence between human and mouse is near the point of optimal value for dual-genome gene prediction [9-11].

Twinscan is one of the most accurate *de novo *dual-genome gene prediction algorithms. It has proven effective for genome annotation in nematodes [12], plants [13], fungi [14], and mammals [6,15]. Recently, the gene-prediction program N-SCAN was introduced as a way to incorporate whole-genome multiple alignments into gene prediction [11]. Twinscan is a special case of the more general N-SCAN algorithm.

Both Twinscan and N-SCAN have focused on the prediction of the single mostly likely transcript in a given gene locus, although alternative splicing is now known to occur in a large majority of mammalian genes. In fact, Kan *et al*. [16] reported that nearly all genes with high expressed sequence tag (EST) coverage showed evidence of multiple splice forms. Even the well characterized human alpha globin cluster was recently shown to contain previously unknown, small, alternatively spliced exons [17]. Moreover, rare alternatively spliced transcripts can have important consequences in health and disease [18].

In an attempt both to address the problem of *de novo *prediction of alternatively spliced genes and to improve multi-genome *de novo *gene prediction, we developed the MARS ('Multiple Informants: Alternative Splices') extension to the Twinscan algorithm.

Almost all current methods for automatically annotating alternatively spliced transcripts rely on a rich EST database [19-21]. One of the few exceptions to an EST-based technique used a pair-hidden Markov model (pair-HMM) to successfully identify alternatively spliced exons conserved in human and mouse [22]. These conserved alternative splicing events are thought to be relatively rare [23]. MARS seeks to leverage the apparently more common situation that for some human genes only one splice variant appears to be conserved in another species [24,25]. One recently described example is the *Tfam *gene, which encodes a mitochondrial transcription factor and has a conserved alternative isoform in primates and rat, but not in mouse [26].

### Description of the MARS algorithm

The MARS algorithm consists of two major steps. In the first step, transcript predictions are created from a number of different evolutionarily related informant sequences using Twinscan. For EGASP, MARS used the publicly available assemblies of the mouse (UCSC id mm5), rat (rn3), dog (canFam1), chicken (galGal1), frog (xenTro1), and opossum (monDom1) genomes as informant sources for Twinscan. These six informant sources make up the informant set. In the second step of the algorithm, the predicted transcripts based on each of the informant sources in the informant set are collected into multi-transcript genes using coding (CDS) region overlap. We refer to gene predictions created this way as MARS genes. MARS genes may be created from any informant subset that contains two or more informant sources.

The predictions described in this paper are based on a version of the MARS algorithm that has been updated compared to the version of the algorithm used to create the predictions submitted to the EGASP workshop. The current predictions use each member of the informant set as a pairwise informant sequence for Twinscan, which is run once for each of the sequences in the informant set to generate transcript predictions based on each specific informant sequence (for example, a total of six times for the informant set described above). This set of transcript predictions is collected into MARS genes.

For the predictions submitted to the EGASP workshop and used in the official evaluation [1], the first step transcript predictions were based on probabilistic combinations of the mouse conservation model with the conservation model from each of the other informant sequences [27]. Briefly, this strategy defines a weighted average of the mouse conservation model with the conservation model of another informant source within the Twinscan probability model to produce the single best transcript predictions based on both informant sources simultaneously. We refer to this procedure as the 'full weight' method, and it is described in detail elsewhere [27]. Thus, the EGASP submissions were created from a set of transcripts based on running Twinscan five times with uniformly weighted averages of the probability models for mouse-rat, mouse-dog, mouse-chicken, mouse-frog, and mouse-opossum. This set of transcripts was collected into MARS genes as described above.

MARS currently predicts only the coding (CDS) regions of genes, thus all references to exons and transcripts are to coding exons and coding transcripts only.

## Results

The results for the updated MARS algorithm differ from those reported in the EGASP summary because of the updates to the MARS algorithm that are described above. Compared to the submitted predictions, those produced from the updated MARS algorithm are more sensitive compared to the GENCODE annotation, but less specific at both the transcript and exon levels. A summary of the accuracy of the EGASP submission version of the MARS algorithm and the updated version described in this paper is given in Table 1. The updated predictions also include approximately twice as many coding transcripts per gene as the predictions submitted to EGASP. Because we made very limited use of the 13 EGASP training regions, we have chosen to present results here based on all 44 regions. These considerations result in a slight difference between the EGASP evaluation of the submitted results and those displayed in Table 1, but do not materially change the results or the interpretation of them.

### Transcript predictions from individual informant sources

For the set of all 44 ENCODE regions, each individual informant results in a similar number of predicted transcripts (Table 2). Informant sources at greater evolutionary distances tend to result in fewer, longer transcripts than informants within the mammalian lineage. However, the summary information from the ENCODE regions presented in Table 2 only hints at the diversity of predicted transcripts from the various informant sources. For example, mouse and rat shared a common ancestor approximately 25 million years ago and align similar fractions of the human genome using our alignment procedure (see Materials and methods and Table 3), but using these two rodent genome sequences as informant sources leads to a significantly different set of transcripts. In fact, the total number of predicted transcripts made using the mouse genome as the informant sequence is similar to the total number of predicted transcripts using the rat genome as the informant sequence (486 and 476, respectively), but less than 50% (213) of these transcripts are predicted to have identical intron-exon structure. Similar results are seen on the human genome as a whole (data not shown).

**Figure 1:**
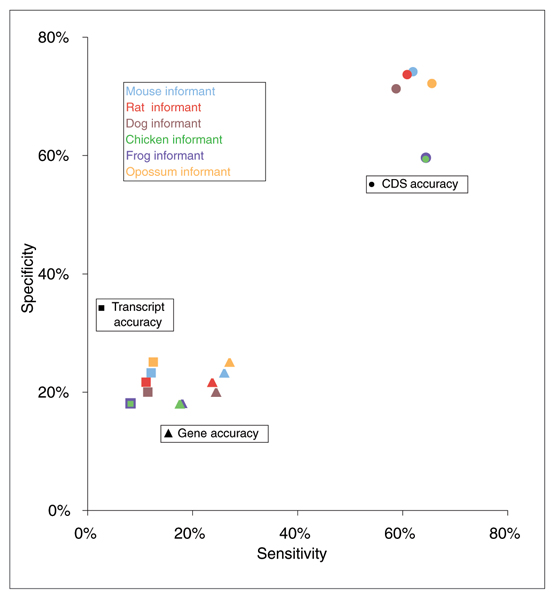
Pair-wise predictive accuracy for each of the six sequences in the informant set. The sensitivity and specificity, as compared to the GENCODE annotations, of Twinscan predictions based on the mouse (blue), rat (red), dog (brown), chicken (green), frog (purple), and opossum (orange) informant sequences. Gene level accuracy (triangles), transcript level accuracy (squares), and coding exon level accuracy (circles) are presented.

The four mammalian informant sequences lead to more accurate predictions than either the frog or the chicken informant. Predictions based on the opossum informant sequence are slightly more accurate than those based on either mouse or rat (Figure 1). Compared to the rodent informant sequences the dog sequence aligns significantly more of the ENCODE regions, without additional alignment in the coding sequence. Conversely, the opossum aligns approximately one-third the total number of bases as the rodent sequences, while retaining alignment in 76% of the coding regions (Table 3).

### Informative value of the pair-wise alignments

**Figure 2:**
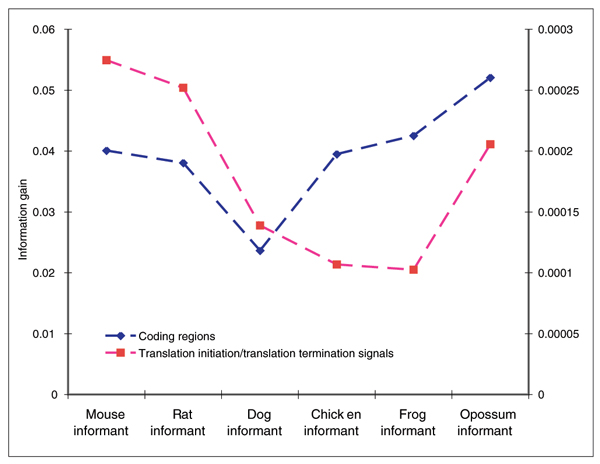
The information gain for the informant alignments with respect to the training set annotations for the six informant sequences. The information gain in the coding portion of the model is displayed in blue with the scale on the left side of the graph. The information gain for the translation initiation and termination signals is displayed in red with the scale on the right-hand side of the graph.

The alignment characteristics for each of the six informant sequences shown in Table 3 are primarily responsible for the characteristics of the pair-wise prediction sets shown in Table 2. To asses how the alignments affect the various components of the Twinscan conservation model, we calculated the information gain of the alignments with respect to the training sequence annotations (see Figure 2 and Materials and methods). The difference in the number of exons per transcript is partially the effect of the amount of information available to the coding portion of the model and the translation initiation and termination signals (that is, the transcript ends). In cases such as mouse, rat, and opossum, where the information gain of the alignments with respect to the annotations is relatively high in both the coding regions and the transcript ends, the number of exons per transcript most closely resembles the annotation. When the information gain for the coding region portion of the conservation model is relatively high and the information gain for the transcript ends is relatively low, longer genes are predicted because the relative information gain of correct gene boundaries is low with respect to incorrect gene boundaries, thus the model is less inclined to end a transcript. In other words, for the case of the frog and the chicken informant sequence, it is more probable, under the model, for a gene to contain additional internal exons rather than boundary exons, which also contain the translation initiation or translation termination signals. This effect also leads to a greater number of exon predictions for the more distantly related informant species. For the case of the dog informant, in which the information gain in both the coding regions and the transcript ends of the model is relatively low, genes are predicted with fewer exons than the annotation. The number of exons per transcript from the dog informant-based predictions is more similar to *ab initio *transcript predictions that do not use evolutionary conservation, such as those reported in group 2 of the EGASP experiment [1].

### MARS genes predicted from informant sets

**Figure 3:**
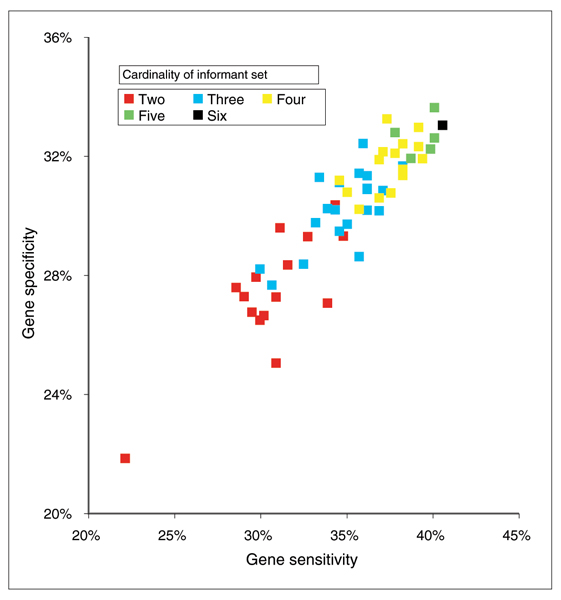
Gene accuracy versus informant subset size. The effect of informant subset size on gene level sensitivity and specificity compared to the GENCODE annotations.

As MARS genes are created from an increasing number of informant sources, we see an increase in predictive sensitivity as the transcripts based on each additional informant sequence are added to the genes. At the same time, the gene specificity improves as addition of longer transcripts from non-mammalian informant sources leads to longer genes (Figure 3).

The predictive sensitivity of both the coding exons and complete coding transcripts also increases as the predictions based on each additional informant sequence are clustered together, but the specificity falls as the number of apparent false positive transcripts and CDS exons increases. The difference in the performance trend at the gene level and the transcript level is based on the definition of gene level accuracy, which rewards predicting at least one transcript correctly with no penalty for additional, incorrectly predicted transcripts.

Both the number of coding exons and the number of transcripts in each MARS gene increase with the size of the informant set (Table 4). This increase corresponds partly to the usage of distantly related informant species in the informant set. For example, MARS genes predicted by the three species informant subset that includes mouse, rat, and dog average 8.07 exons per gene, while MARS genes from the frog, chicken, and opossum informant subset average 11.64 exons per gene.

We separately evaluated the subset of transcripts that are predicted based on at least two informant sequences. These transcripts are significantly more specific at all levels of the evaluation (Table 5), although the predictions are less sensitive (as expected). The set of transcripts common to only mammalian informant sources is more specific than the set common to all informant sources and only slightly less sensitive (Table 5).

### Prediction of alternatively spliced transcripts

Using the informant set consisting of all six informant sources, MARS correctly predicts alternatively spliced transcripts for 11 of the 236 multi-exon GENCODE genes that are alternatively spliced in the coding region of their transcripts. For these 11 genes, a total of 24 (out of 59) correct transcripts are predicted (we observed that just 2 of these 11 genes accounted for 25 of the 59 coding transcripts: RP1-309K30.2 on ENr333 and RP4-696P19.3 on ENr334). Moreover, when compared to a set of 134 cassette (that is, skipped) coding exons from the GENCODE annotation, MARS predicted 85 of these exons correctly in at least one transcript, including 19 that are correctly predicted as cassette exons. MARS predicts a total of 247 cassette exons.

When all six informant sources are used simultaneously, the predictive sensitivity is at its highest. MARS predicts about twice as many unique coding transcripts (1,873) as exist in the reference GENCODE annotation (975).

### Experimental verification

An important part of the EGASP experiment is the attempt to experimentally validate a subset of the computational predictions outside of the reference GENCODE annotation. As part of EGASP, Guigo *et al*. [1] selected a total of 47 exon pairs predicted by MARS for experimental confirmation by RT-PCR. Of these, 7 (15%) were found to be expressed in at least one tissue. Interestingly, although a number of the other EGASP gene-prediction methods also predicted as many as 4 of these exon pairs, these 7 were the only ones that could be confirmed in the EGASP experiment.

## Discussion

One of the goals of the ENCODE pilot project was to develop new high-throughput methods to identify the functional elements in the human genome [28,29]. To address the continued need for *de novo *gene discovery, we have introduced the MARS method for prediction of alternatively spliced transcripts without the use of any expressed sequence information. MARS genes are built by combining the predicted transcripts from a number of informant species and are significantly more likely to contain correctly predicted transcripts than any individual informant. MARS performed effectively when compared to other dual-genome *de novo *gene prediction systems in EGASP [1] and is unique among the EGASP methods in its ability to predict alternatively spliced transcripts using only patterns found in pair-wise alignments between a target sequence and a set of informant sequences.

We have updated the MARS algorithm between the EGASP workshop and submission of this paper. The updated version of MARS correctly predicts multiple alternatively spliced transcripts at one additional locus compared to the submitted version. Additionally, the updated algorithm is more sensitive for all measures, although this increased sensitivity comes at a cost of a significant reduction in specificity at the transcript and exon levels compared to the version submitted to the EGASP workshop. Regardless, we feel the update is justified on theoretical grounds because the original submission gives too much consideration to the mouse informant to the detriment of other informant sources. A second source of error comes from the addition of transcripts from the two non-mammalian informant sources, which appears to have enriched the prediction set for false positive transcripts.

A number of gene-finding algorithms create consensus genes by combining sets of gene predictions and other information [13,30]. One example in the EGASP experiment is JIGSAW [31], a program that uses 'any information' (EGASP category 1) to create gene structures. Much of the information used by JIGSAW is based on expressed sequences and is, therefore, not directly comparable to the EGASP dual-genome (category 4) predictions. Because MARS genes are created by overlapping transcript predictions from a number of sources, we were interested to see if these transcript predictions could be statistically combined to produce more accurate consensus gene structures. To directly address this question, we compared the MARS genes to consensus genes produced by GLEAN, a new gene-prediction algorithm that uses dynamic programming to discover gene structures that maximize the probability of several sources of evidence (A Mackey, personal communication). GLEAN was run using the transcript predictions from the six individual pair-wise sets (mouse, rat, chicken, dog, frog, and opossum) as its only input sources of evidence, although the transcript sets cannot be considered independent sources of information and thus represent a non-traditional use of the algorithm likely to reduce its statistical power (A Mackey, personal communication). The GLEAN consensus predictions at the gene and transcript levels were similar to predictions based on either the rat or dog informant only (that is, less sensitive and specific than the mouse or opossum informant, but more sensitive and specific than the chicken or frog informants). For coding exons, the GLEAN consensus predictions are more sensitive than any of the individual informants and less specific than predictions based on mammalian or marsupial informants.

Our analysis of the information in the pair-wise alignments shows that some characteristics of the transcript predictions are a consequence of the alignments themselves. Importantly, the concentration of alignments from the opossum in the coding sequences of the ENCODE regions and the pairwise predictive accuracy of the opossum informant show that the draft genome sequence of *Monodelphis domestica *is already a valuable tool for dual-genome gene prediction. A more complete or even finished opossum assembly could prove especially powerful for annotating the functional regions in the human genome.

The MARS method is computationally tractable with computational requirements, growing essentially linearly with the number of informant sequences and it can take advantage of additional genome sequences as they become available without extensive reanalysis. Other methods for annotating alternatively spliced transcripts are generally based on information from expressed sequences; thus, the annotations produced are experimentally supported. MARS genes, in general, do not have such support and thus provide a potential pool for experimental validation of novel splice forms [32,33].

Recent reports indicate that alternatively spliced exons have specific sequence features associated with them, such as exonic splicing enhancers (ESEs) and exonic splicing silencers (ESSs) [22]. Moreover, some alternatively spliced exons have conservation patterns unlike constitutively spliced exons [34]. Neither of these observations have been incorporated into the MARS model and doing so could lead to more accurate prediction of alternatively spliced transcripts that are not yet supported in EST databases.

## Conclusion

The MARS algorithm is able to predict alternatively spliced transcripts without the use of expressed sequence information, although the number of loci in which multiple predicted transcripts match multiple alternatively spliced transcripts in the GENCODE annotation is relatively small. Based on the current GENCODE annotation, it seems unlikely the majority of alternatively spliced transcripts predicted by MARS are actually produced. However, the results of the EGASP experimental validation of novel predictions show that among the EGASP entries, more MARS predictions were confirmed than for any other method [1]. These results are consistent with the previous reannotation of chromosome 22 in light of additional data that resulted in a significant number of new annotations, including many alternatively spliced transcripts [35]. Finally, the large fraction of incomplete transcripts in the current GENCODE annotation suggests that we are still some distance from finished annotation.

We propose that the selection of other novel alternative transcripts for experimental confirmation may be guided by looking first to those transcripts predicted with identical structure using several informant sequences. In fact, the set of 449 complete transcripts that is common to more than one informant source is approximately three times more specific than the complete set of MARS transcripts.

## Materials and methods

### Sequences

All predictions were made on the ENCODE regions as mapped to NCBI Build 35 (UCSC id hg17) of the human genome [36] downloaded from the UCSC genome browser [37,38] on 3 June 2004. The human genome was masked for interspersed, but not low-complexity, repeats using RepeatMasker tables provided by UCSC.

Where possible, each ENCODE region was padded on each side with 750,000 base-pairs (bp) of genomic sequence from the corresponding chromosome to ensure that the predictions were made in true genomic context and because genes were expected to extend beyond the boundaries of the ENCODE regions. The size of the sequence context was chosen based on the memory usage of Twinscan. Restricting the input sequences to the exact boundaries of the ENCODE regions results in a small decrease in predictive accuracy of approximately 1% for all evaluation measures due to incorrectly truncated genes. Informant genome sequences were also downloaded from the UCSC Genome Brower. This set included NCBI Build 33 (UCSC id mm5) of the mouse genome sequence, the canFam1 assembly of the dog genome sequence, the monDom1 assembly of the opossum genome, the rn3 assembly of the rat genome, assembly galGal2 of the chicken genome, and assembly xenTro1 of the frog genome.

### Twinscan version and training set

The results in this study use the TwinscanΦ executable [27] which is an updated version of Twinscan 1.1 [6]. Conservation parameters were trained separately for each of the six informant species on a set of 3,072 human RefSeq transcripts from 2,477 loci. Genes in the training set are spread across 112 one megabase fragments of the human genome and selected based on characteristics of the genes on the fragments, including gene density and gene length. These conservation parameters are optimized for accurate whole genome predictive accuracy. The training sequences and annotations are available at [39].

The 13 ENCODE training regions provided in advance of the EGASP submission were used only to determine the optimal size and members of the informant set.

### Alignments

All alignments were done with WU-BLAST version 07-14-2004 [40] using a two-stage serial BLAST strategy [41]. First stage BLAST parameters were set at M = 1 N = -1 Q = 5 R = 1 Z = 3000000000 Y = 3000000000 B = 10000 V = 100 W = 11 X = 30 S = 30 S2 = 30 gapS2 = 30 topcomboN = 1. Second stage BLAST alignments used the following more stringent parameters: W = 8 X = 20 S = 15 S2 = 15 topcomboN = 3. For human-chicken and human-frog alignments, Z = 1000000000 was used. The seg and dust filters were used for all alignments. BLAST databases were prepared as previously described [6].

### Information gain calculation

We calculated the information gain for each of the informant sources using our training set by subtracting the 0th order conditional uncertainty of the annotation given the conservation sequence from the annotation uncertainty as follows:

*IG*_*i *_= *H*(*C*_*m*_) - *H*(*C*_*m*_|*A*)

where

*H*(*C*_*m*_) = -*Pr*(*C*_*m*_) × log2*Pr*(*C*_*m*_)

and

*H*(*C*_*m*_|*A*) = -Pr(*M*) (Pr(*c*_*m*_|*M*) log2 Pr(*c*_*m*_|*M*) + Pr(*n*_*m*_|*M*) log2 Pr(*n*_*m*_|*M*))

-Pr(*G*) (Pr(*c*_*m*_|*G*) log2 Pr(*c*_*m*_|*G*) + Pr(*n*_*m*_|*G*) log2 Pr(*n*_*m*_|*G*))

-Pr(*U*) (Pr(*c*_*m*_|*U*) log2 Pr(*c*_*m*_|*U*) + Pr(*n*_*m*_|*U*) log2 Pr(*n*_*m*_|*U*))

Here *C*_*m *_is defined as a random variable representing whether a given base in the genome should be classified as part of the given portion of the Twinscan conservation model (*C*_*m*_) or as not a part of the given portion of the model (*n*_*m*_). We use the maximum likelihood estimate of *C*_*m *_from our training set. For the conditional uncertainty calculation, we condition the probability of *C*_*m *_based on whether the corresponding conservation symbol from the given informant sequence is (*M*)atch, (*G*)ap/mismatch, or (*U*)naligned. In this analysis here we use m ∈ {coding, translation initiation and translation termination signal} portions of the Twinscan conservation model [2].

### Evaluation method

All evaluations were performed as described [1] using the GENCODE annotations as a reference.

### Availability

MARS source code is available on an open source license. All predictions, training materials, and source code are available at the MARS website [39].

## Figures and Tables

**Table 1:** Submitted versus updated prediction characteristics

	ESn	ESp	TSn	TSp	GSn	GSp
Predictions submitted to EGASP	69.3%	65.8%	18.2%	17.8%	38.0%	28.3%
Updated MARS algorithm	74.4%	45.1%	19.4%	10.4%	40.6%	33.0%

A comparison of the predictive accuracy for the MARS genes submitted to the EGASP workshop and those produced by the updated MARS algorithm. The columns are sensitivity and specificity at the coding exon (ESn/ESp), coding transcript (TSn/TSp), and gene level (GSn/GSp).

**Table 2:** Pair-wise prediction characteristics

	Mouse	Rat	Dog	Chicken	Frog	Opossum
Predicted transcripts	486	476	530	431	422	467
Exons per transcript	7.55	7.62	6.82	11.02	11.28	8.54

The total number of predicted transcripts in the 44 ENCODE regions and the number of coding exons per transcript for each of the six informant sources in the MARS informant set.

**Table 3:** Aligned fraction of the ENCODE regions

	Mouse	Rat	Dog	Chicken	Frog	Opossum
Whole regions	15.2%	14.7%	28.8%	2.8%	2.0%	5.6%
Coding sequence	87.5%	85.0%	87.4%	53.0%	50.1%	76.1%

A comparison of the total fraction of bases aligned in the 44 ENCODE regions and the fraction of bases aligned in the coding portion of the GENCODE annotation for each of the informant sources in the MARS informant set. See Materials and methods for the alignment protocol.

**Table 4:** Effect of increasing informant subset size

	Pair-wise prediction characteristics					
	
Informant set size	Two	Three	Four	Five	Six	Annotation
Average transcripts per gene	1.76	2.44	3.05	3.62	4.15	2.25
Average exons per transcript	9.31	9.67	9.94	10.16	10.35	8.64

The number of coding transcripts per gene and coding exons per transcripts increases with the cardinality of the informant set. The gene level accuracy also increases with informant set size (see Figure 3).

**Table 5:** Transcripts common to several informant sources

	Prediction accuracy for transcripts common to several informants					
	
	ESn	ESp	TSn	TSp	GSn	GSp
All transcripts	74.4%	45.1%	19.4%	10.4%	40.6%	33.0%
Common transcripts	43.0%	71.6%	15.1%	29.6%	32.0%	35.0%
Mammalian transcripts	40.0%	77.1%	14.8%	33.7%	31.3%	37.7%

A comparison of the predictive accuracy for all MARS genes, with those having at least two transcripts predicted with identical structure from more than one informant source across the entire informant set, and with those having two transcripts with identical structures from at least two mammalian informant sources. Columns are defined as in Table 1.
